# The effect of ultrasonic pre-treatment on the catalytic activity of lipases in aqueous and non-aqueous media

**DOI:** 10.1186/1752-153X-2-1

**Published:** 2008-01-30

**Authors:** Shweta Shah, Munishwar Nath Gupta

**Affiliations:** 1 Chemistry Department, Indian Institute of Technology Delhi, Hauz Khas, New Delhi 110016, India

## Abstract

**Background:**

Ultrasound has been used to accelerate the rates of numerous chemical reactions, however its effects on enzymatic reactions have been less extensively studied. While known to result in the acceleration of enzyme-catalysed reactions, ultrasonication has also been shown to induce enzyme inactivation. In this study we investigated the effects of ultrasonic pretreatment on lipases in both aqueous and non-aqueous media.

**Results:**

Our results show that the ultrasonic pre-irradiation of lipases (from *Burkholderia cepacia *and *Pseudomonas fluorescens*) in aqueous buffer and organic solvents enhanced enzymic activities. In addition, we report the enhancement of hydrolytic (esterase) and transesterification activities.

On using pre-irradiated enzyme, we found that the conversion rate for the transesterification of ethyl butyrate to butyl butyrate, increased from 66% to 82%. Similarly, a 79% conversion of *Jatropha *oil to biodiesel was observed upon employing pre-irradiated enzyme, in contrast to a 34% conversion with untreated enzyme.

CD spectra showed that while the enzyme's secondary structure remained largely unaffected, the microenvironments of aromatic amino acids were altered, with perturbation of the tertiary structure having also occurred. SEM analysis demonstrated significant morphological changes in the enzyme preparation as a result of ultrasonication.

**Conclusion:**

In contrast to the effects of ultrasonic irradiation on other enzymes, for the lipases focused upon in this study, we report an enhancement of biocatalytic activity, which is thought to originate from morphological changes on the macro and molecular levels.

## Background

Ultrasound has been used to accelerate the rates of numerous of chemical reactions [1-3]. These rate enhancements, mediated by cavitation, are believed to originate from the build up of high local pressures (up to 1000 atm) and temperatures (up to 5000 K), as well as increased catalytic surface areas.

The effects of ultrasound on enzymatic reactions, however, have been less extensively studied [4-9]. The few studies that have been carried out can be categorised into two main groups. The first approach involves using ultrasound as an enzymic pretreatment to reduce particle size. This is especially relevant when using enzyme powders to catalyse reactions in organic media [10-12]. In such cases, the reduction in particle size and consequent increase in the catalytic surface area are thought to reduce mass transfer limitations. The second approach involves the use of ultrasound throughout the reaction. Here the cavitation energy is thought to accelerate the reaction rate, yet the mechanism by which this occurs is unclear. It may be that by increasing the movement of liquid molecules, the substrate's access to the active site is increased. Other mechanisms have also been suggested [5].

While it has been shown that the second of these approaches can accelerate enzymatic reactions [4,6], other reports have demonstrated enzyme inactivation [7-9]. In general, enzymes are known to be more stable in nearly-anhydrous organic solvents [10-12], therefore it is not surprising that all the reported cases of rate enhancements resulting from ultrasonic treatment are those involving enzymic catalysis in organic media [4,6].

By focusing on lipases, which have extensive applications in biotransformations [10-12], the aim of this study was to look at the effect of ultrasonic pretreatment on catalytic performance.

We placed lipases in both aqueous as well as organic media during ultrasonic irradiation. The pretreated lipases were evaluated for both esterase (in water) and transesterification (in organic solvent) activities. Lastly, ultrasonically pre-irradiated lipase was also used in the transesterification of *Jatropha *oil to biodiesel. Biodiesel production is an interesting application of lipase-catalysed transesterification [13,14], for which *Jatropha *oil can be considered an economically sound choice as a starting material [14,15].

## Results and Discussion

### The effect of ultrasonic pre-irradiation on the esterase activity of lipases

**Figure 1:**
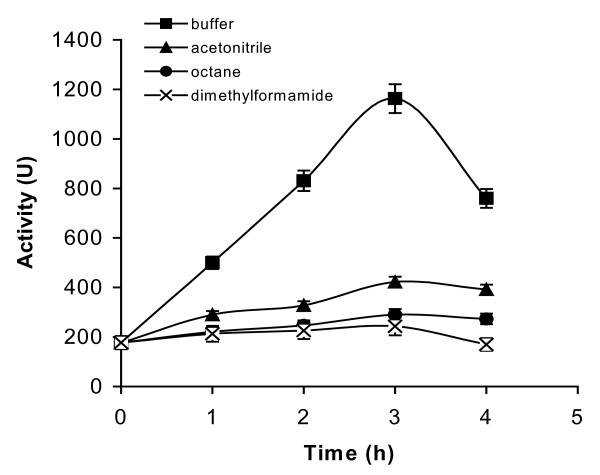
**The effect of ultrasonic pre-irradiation on the stability of *Burkholderia cepacia *lipase in various solvents**. pH tuned *Burkholderia cepacia *lipase (1 mg) was suspended in different solvents (100 μl each) and ultrasonicated at 110W continually. In this study, we carried out ultrasonication for 5 minutes, followed by a break of 10 minutes between each cycle. This ensured that the temperature of the sample could be maintained at 40 ± 1°C throughout. The value on the x-axis denotes the total time during which the ultrasonicator was 'on'. Samples were extracted at various intervals (i.e. 1, 2, 3, and 4 h) and an esterase assay was performed using *p*NPP as a substrate. The experiments were carried out in triplicate. The error bar reflects the reproducibility for each data point.

As expected, continuous ultrasonic pre-irradiation of the enzyme solution/suspension led to an increase in temperature. In order to assess the thermal effect (as a stress factor for denaturation), we decided to expose the enzyme solution/suspension to ultrasound for 5 min at 40°C, before maintaining the solution/suspension at 40°C in an incubator for a further 10 min. This method of interrupted ultrasonic pre-irradiation succeeded in maintaining the sample at 40°C ± 1. Figure 1 outlines the effect of ultrasonic pre-irradiation on the stability of *Burkholderia cepacia *lipase in aqueous buffer (20 mM phosphate buffer, pH 7.0) and three different organic solvents of differing polarities. The stability was evaluated by taking aliquots periodically and assaying the esterase activity in aqueous media.

For enzymes suspended in organic solvents, the latter was removed by centrifugation. The centrifuged enzyme was then transferred to the aqueous assay (hydrolysis of *p*-nitrophenyl palmitate) system in which it dissolved as expected (note: there was no organic solvent present in the aqueous assay system). In all cases, enzymic activity was seen initially to increase before then decreasing as a result of ultrasonic pre-irradiation (Figure 1). The effects were marginal for enzyme suspensions in organic solvents, but were quite significant for those in aqueous solution. The likeliest explanation for the trend seems to be that pretreatment caused a decrease in the particle size of the catalyst, with further treatment then inducing enzyme inactivation. It is noteworthy that for none of the solvents, except DMF (the most polar organic solvent), was the final activity (after ultrasonication for 4 h) less than the starting activity (without ultrasonication pretreatment).

Controls were also carried out in which the *Burkholderia cepacia *lipase was incubated for 4 h at 40°C (in a water bath, without irradiation) in various solvents. Here surviving activities of 75%, 100%, 100% and 53% for aqueous buffer, acetonitrile, octane and DMF respectively (data not shown) were obtained. Therefore ultrasonic pretreatment compensated effectively for thermal inactivation, with pretreatment in aqueous buffer being more effective, in terms of observed enzyme activity, than in organic solvents. Presumably, cavitation energy and its dispersion differ in aqueous and organic media.

It is relevant to compare these results with those reported by Vulfson *et al *[4] on subtilisin-catalysed transesterification. In that study a similar power output was employed (150 W), with the temperature maintained using a thermostatically-controlled glass reaction vessel. Ultrasonic treatment of subtilisin in phosphate buffer led to inactivation of the enzyme (50% in around out 2 h), while no equivalent effect was observed in t-amyl alcohol (containing 1% vv^-1 ^buffer). Sinisterra [5] has also reported that the ultrasonic inactivation of subtilisin is more pronounced in aqueous media than under biphasic conditions (*t*-amyl alcohol, 1% phosphate buffer).

Another study of interest is that of Özbek and Ulgen [8], who recently investigated the effect of ultrasound on six enzymes (four dehydrogenases, alkaline phosphatase and β-galactosidase) in aqueous buffers. Apart from alkaline phosphatase, all other enzymes showed (variable) inactivation profiles (although ultrasonic treatment was carried out at 5°C). Higher ultrasonication times or power outputs resulted in greater inactivation. In addition, it was observed that on increasing the viscosity of the media by addition of glycerol increased the ultrasonic inactivation. Interestingly, none of these three studies reported enzyme activation. However, two studies by Ishimori *et al. *and [16] Sakakibara *et al. *[17], do report enhanced reaction rates resulting from the application of ultrasound to enzymatic reactions in aqueous buffers. The first of these reported the acceleration of α-chymotrypsin activity, whilst the second demonstrated that the activity of invertase was promoted by ultrasound at low substrate concentration. While the V_max _remained unaltered, K_m _roughly halved upon ultrasonication (i.e. affinity for the substrate increased).

Thus to recapitulate, as indicated by figure 1, ultrasonic pretreatment resulted in the aqueous enzyme being more active in aqueous buffer. These data are unusual and have not been previously observed.

**Figure 2:**
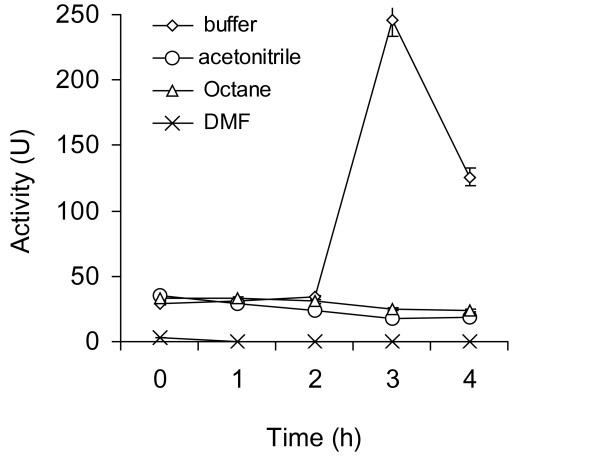
**The effect of ultrasonic pre-irradiation on the stability of *Pseudomonas fluorescens *lipase in various solvents**. pH tuned *Pseudomonas fluorescens *lipase (1 mg) was suspended in different solvents (100 μl each) and ultrasonicated at 110W periodically. In this study, we carried out ultrasonication for 5 minutes, followed by a break of 10 minutes between each cycle. This ensured that the temperature of the sample could be maintained at 40 ± 1°C throughout. The value on the x-axis denotes the total time during which the ultrasonicator was 'on'. Samples were extracted at various intervals (i.e. 1, 2, 3, and 4 h) and an esterase assay was performed out using *p*NPP as a substrate. The experiments were carried out in triplicate. The error bar reflects the reproducibility for each data point.

**Figure 3:**
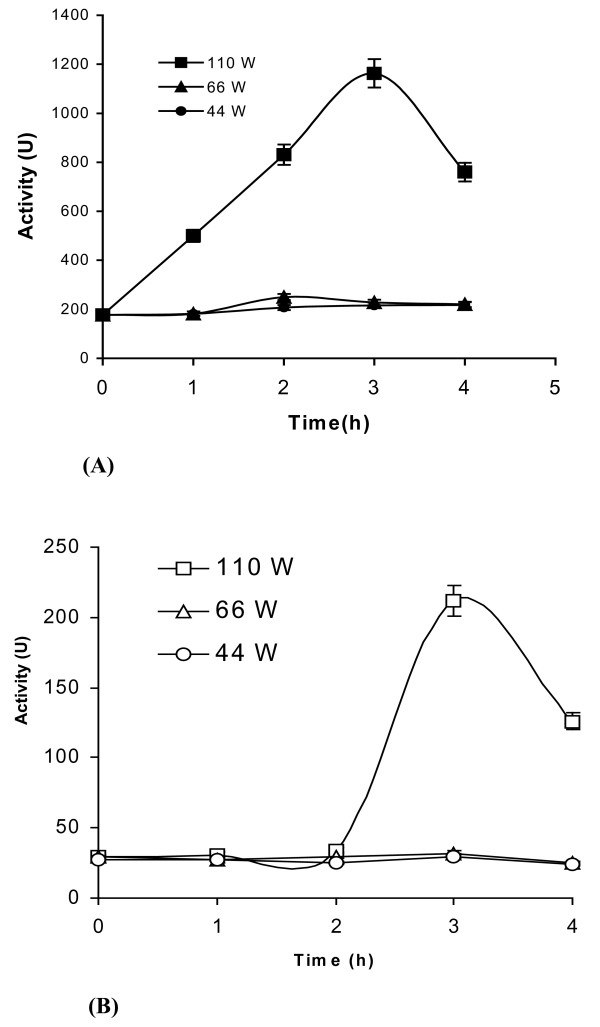
**a and b. The effect of ultrasonic pre-irradiation (at different power ratings) on lipase stability in aqueous media**. pH tuned lipase (1 mg) in 0.1 M sodium phosphate buffer, pH 7.0 (100 μl) was sonicated at various power ratings (i.e 110 W, 66 W, 44 W) periodically. In this study, we carried out ultrasonication for 5 minutes, followed by a break of 10 minutes between each cycle. This ensured that the temperature of the sample could be maintained at 40 ± 1°C throughout. The value on the x-axis denotes the total time during which the ultrasonicator was 'on'. Samples were extracted at various intervals (i.e. 1, 2, 3, and 4 h) and an esterase assay was performed using pNPP as a substrate. **Note**: (a) *Burkholderia cepacia *lipase and (b) *Pseudomonas fluorescens *lipase. The experiments were carried out in triplicate. The error bar reflects the reproducibility for each data point.

**Figure 4:**
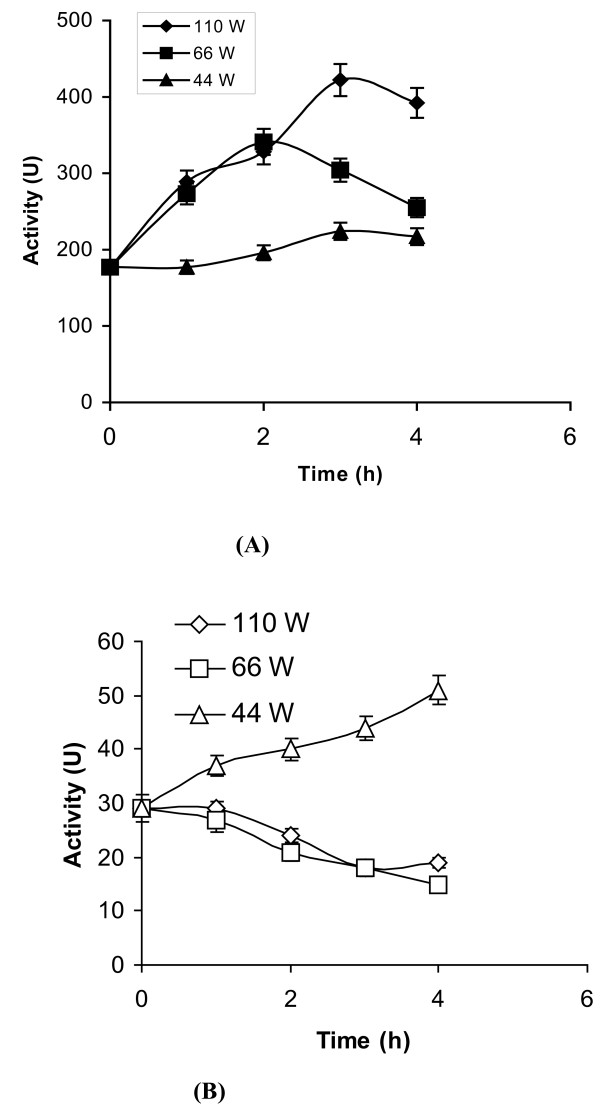
**a and b. The effect of ultrasonic pre-treatment (at various power ratings) on lipase stability in acetonitrile**. pH tuned lipase (1 mg) in acetonitrile (100 μl) was ultrasonicated at various power ratings (i.e. 110 W, 66 W and 44 W) continually. In this study, we carried out ultrasonication for 5 minutes, followed by a break of 10 minutes between each cycle. This ensured that the temperature of the sample could be maintained at 40 ± 1°C throughout. The value on the x-axis denotes the total time during which the ultrasonicator was 'on'. Samples were extracted at various intervals (i.e. 1, 2, 3, and 4 h) and an esterase assay was performed using pNPP as a substrate. **Note**: (a) *Burkholderia cepacia *lipase and (b) *Pseudomonas fluorescens *lipase. The experiments were carried out in triplicate. The error bar reflects the reproducibility for each data point.

Figure 2 outlines the results obtained from a similar study involving the lipase from *Pseudomonas fluorescens*. Here pretreatment in aqueous buffer resulted only in enhancement of enzyme activity. Figure 3a and 3b shows the effect of varying the ultrasonic pre-irradiation power for both enzymes. As can be seen, a reduction in power from 110 W had only a marginal effect. Figure 4a and 4b outlines the results of suspending both enzymes in acetonitrile. With *Burkholderia cepacia *lipase there is a direct correlation between the power output and the enhancement in catalytic activity; employing higher power (i.e. 110W) resulted in maximum enhancement (Figure 4a). However lipase from *Pseudomonas fluorescens *exhibits different behaviour. While irradiation at both 110 and 66Wlowered enzymic activity, pretreatment at 44 W gave only marginal enhancement (Figure 4b).

### The effect of ultrasonic pre-irradiation on lipase activity in organic solvents

**Figure 5:**
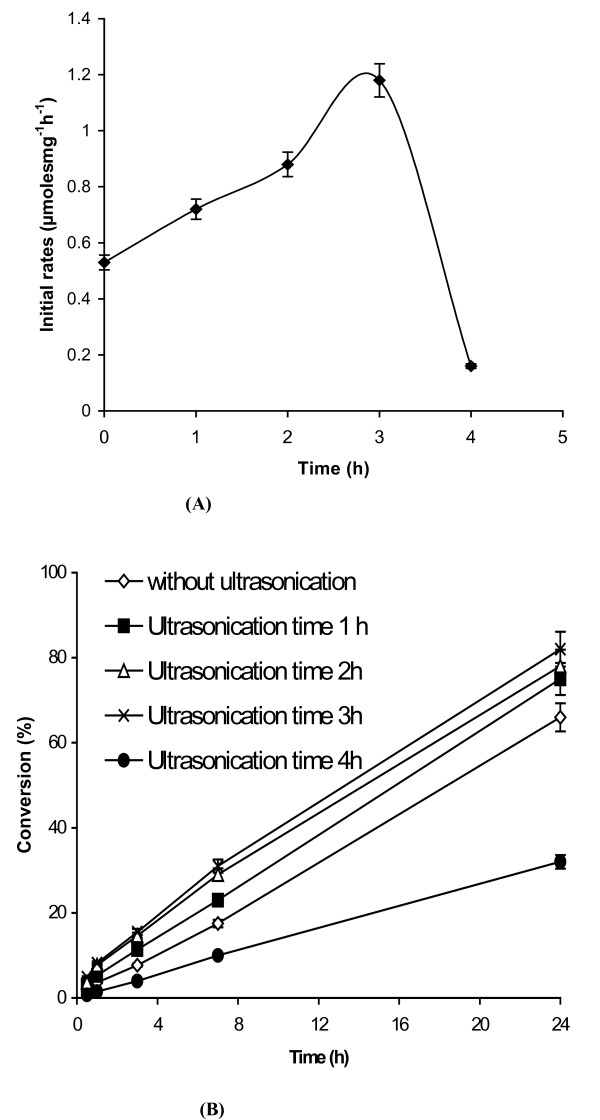
**a and b. The effect of ultrasonic pre-irradiation on B *urkholderia cepacia *lipase in the transesterification of ethyl butyrate with butanol in octane**. EPRPs of ultrasonic pre-irradiated lipase/untreated lipase were prepared and used for the transesterification of ethylbutyrtae with *n*-butanol in *n*-octane as described in the materials and methods section. **Note**: (a) Initial rates of transesterification (b) conversion percentages. The experiments were carried out in triplicate. The error bar reflects the reproducibility for each data point.

We decided to apply our experiences with the ultrasonic pre-irradiation of lipases to a biotransformation in organic media. The transesterification (ethyl butyrate with butanol in anhydrous octane) activity was evaluated after 'drying', that is, removing bulk water from the pre-irradiated enzyme sample. (It has been reported that drying is more efficient when carried out through precipitation with an organic solvent rather than lyophilisation [18,19].) The initial rate of transesterification increased from 0.53 to 1.18 μmoles^-1^mg^-1^min at the optimum pre-irradiation time of 3 h (Figure 5a). The consequence of the enhanced initial rate was also reflected in the percentage conversions obtained (Figure 5b): for enzyme pre-irradiated for 3 h, 82% conversion was obtained over 24 h, whilst untreated enzyme gave 66% conversion under the same conditions, and an oversonicated (for 4 h) sample gave only 32% conversion.

The enhancement of initial enzymic rates is a challenge particularly relevant to industry [10,12]. The transesterification activity of lipases has already been exploited in the synthesis and kinetic resolution of several compounds [10-12]. In order to illustrate this, we decided to look at the production of biodiesel from *Jatropha *oil. Biodiesel is a diesel-equivalent processed fuel consisting of short chain alkyl (methyl or ethyl) esters of fatty acids, which can be used (alone, or blended with conventional diesel fuel) in unmodified diesel-engine vehicles [20]. It is a more environmentally-friendly fuel and its enzymatic preparation has attracted considerable attention [20-22]. The enzymatic route involves the lipase-catalysed tranesterification of plant oils with ethyl/methyl alcohol. The use of *Jatropha *oil as the starting material is favourable, given for instance its inedibility and easy cultivation, even on wasteland [14,15]. Both chemical [14] and enzymic [15] preparations have been described in the literature, and earlier studies have demonstrated the benefit of a solvent-free approach [22,23].

**Figure 6:**
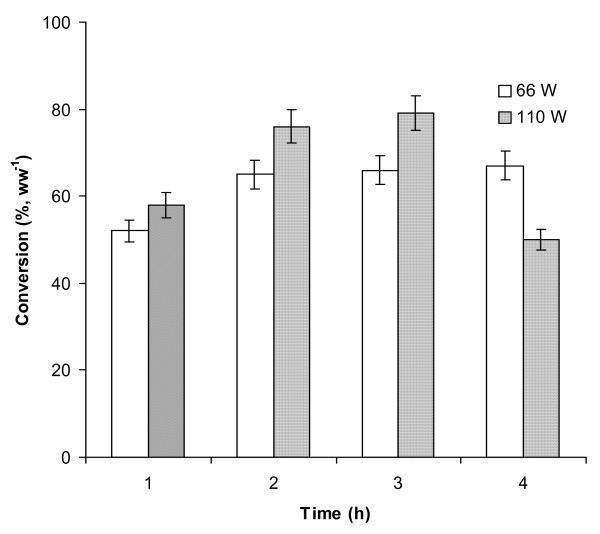
**The effect of ultrasonic pre-irradiation (for various time intervals) on the transesterification activity of *Burkholderia cepacia *lipase with *Jatropha oil***. In a control (without ultrasonicated lipase) a 34% conversion was obtained (data not shown). The experiments were carried out in triplicate. The error bar reflects the reproducibility for each data point.

We decided to carry out pretreatment of lipase in *Jatropha *oil directly before initiating transesterification with the addition of ethyl alcohol. The *Burkholderia cepacia *lipase was chosen owing to its responding better to ultrasonic pre-irradiation. In addition, because outputs of 110 and 66 W were observed to yield better results when using organic solvents, these settings were again adopted (Figure 6). Pretreatment for 2 or 3 h at 110 W gave the optimum result, that is a 79% conversion over 24 h. The untreated enzyme (used as control) gave only 34% conversion over the same time period.

### Structural and morphological changes in the lipase preparation as a result of ultrasonication

**Figure 7:**
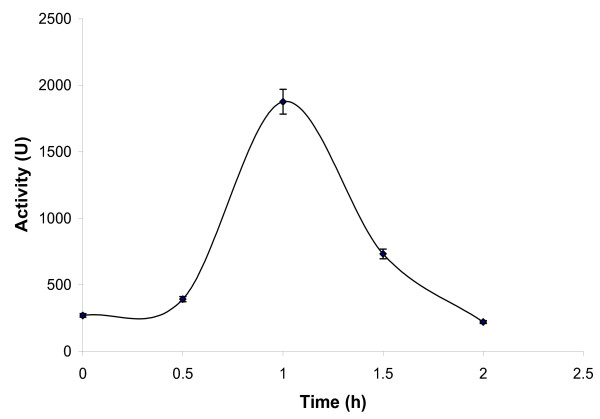
**The effect of ultrasonic pre-irradiation on the stability of purified *Burkholderia cepacia *lipase in buffer**. Purified *Burkholderia cepacia *lipase (366 U) was dissolved in buffer and ultrasonicated at 110W. In this study, we carried out ultrasonication for 5 minutes, followed by a break of 10 minutes between each cycle. This ensured that the temperature of the sample could be maintained at 40 ± 1°C throughout. The value on the x-axis denotes the total time during which the ultrasonicator was 'on'. Samples were extracted at various intervals (i.e. 0.5, 1, 1.5 and 2 h) and an esterase assay was performed using pNPP as a substrate. The experiments were carried out in triplicate. The error bar reflects the reproducibility for each data point.

In order to determine whether pretreatment affected the protein structure, the *Burkholderia cepacia *lipase was purified and the effects of pre-irradiation were investigated (Figure 7). Although no inactivation was observed, the pure protein was most affected, with maximum activity resulting after pre-irradiation for 1 h.

**Figure 8:**
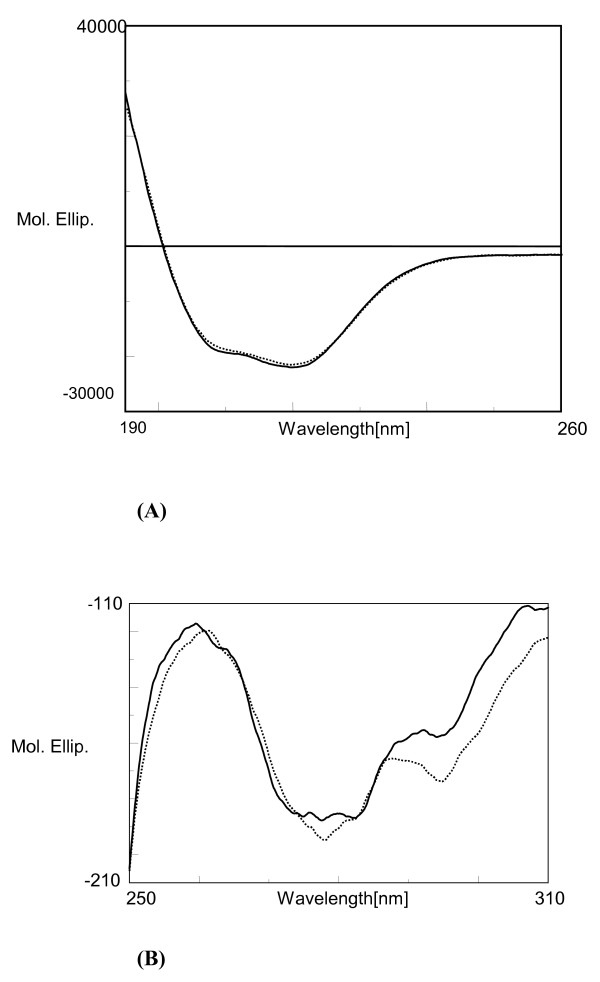
(a) Far-UV CD spectra of *Burkholderia cepacia *lipase; (b) Near-UV CD spectra of *Burkholderia cepacia *lipase.

The far-UV CD spectra of the untreated and irradiated samples were identical, indicating that ultrasonic irradiation had not affected the lipase's secondary structure (figure 8a). However two differences could be seen in the 250–300 nm spectral region (Figure 8b). Signals in the near-UV CD spectral region are diagnostic of the microenvironments of aromatic residues phenylalanine, tyrosine and tryptophan. Signals in the 250–270 nm region are attributable to phenylalanine, with signals in the 270–290 nm region attributable to tyrosine, and those in the 280–300 region indicate the presence of tryptophan [24]. The CD spectra (Figure 8b) showed that ultrasonic pre-irradiation led to perturbation of the tyrosine and tryptophan environments. Ultrasonic pre-irradiation enhanced the negative band intensity, indicating slight perturbation of the tertiary structure [24].

**Figure 9:**
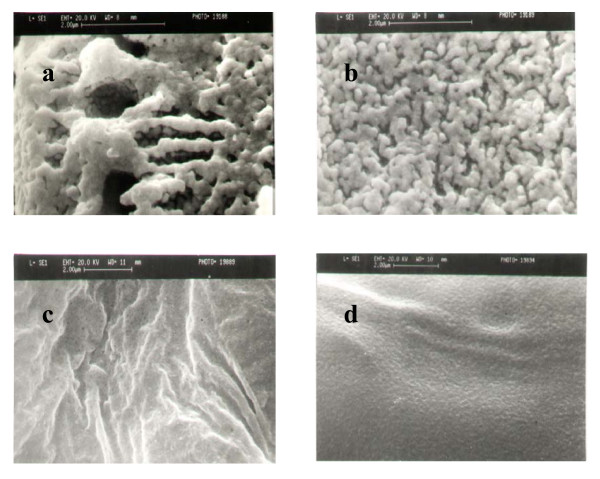
Scanning electron micrographs of (a) *Burkholderia cepacia *lipase without ultrasonication (directly taken from bottle); (b) *Burkholderia cepacia *lipase (directly taken from bottle) after ultrasonication; (c) purified *Burkholderia cepacia *lipase without ultrasonication; and (d) purified *Burkholderia *cepacia lipase after ultrasonication. The bar represents 2 μm.

The SEM of the untreated and irradiated lipase samples (Figure 9) showed that definite morphological changes were brought about by ultrasonication. Initially, the preparation was more or less monolithic in nature, however, following pre-treatment, small spheres or, in the case of pure enzyme, a fine powder could be observed on the surface. This is thought to have increased the surface area of the catalyst. We can therefore conclude that activation of the lipase by ultrasonic irradiation originated both in morphological and structural changes at the molecular level.

## Conclusion

Limited data on the effects of ultrasonication on enzyme activity are available in the literature. While in some cases, ultrasonication results in the loss of enzymic activity, in others it leads to the enhancement of the reaction rate. In this study we demonstrated that ultrasonic pretreatment enhanced enzyme activity in water and organic media.

## Experimental

*Jatropha *oil was obtained from Dr. Jayaveera, Jawaharlal Nehru Technological University Oil Technological Research Institute, Anatapur, India. *Burkholderia cepacia *(PS) and *Pseudomonas fluorescens *(AK) were kind gifts from Amano Enzyme Inc., Nagoya, Japan. p-Nitrophenylpalmitate (pNPP) and Sephadex G-75 were bought from Sigma Chemical Co., St Louis, USA. All solvents were of anhydrous grade and were obtained from J. T. Baker, USA. They were further dried by being gentle shaken with 3 Å molecular sieves (E. Merck, Mumbai, India).

### Enzyme preparations

Lipase from *Burkholderia cepacia *(50 mg) was dissolved in 0.5 ml of 20 mM sodium phosphate buffer (pH 7.0). *Pseudomonas fluorescens *lipase (50 mg) was dissolved in 0.5 ml of 20 mM sodium phosphate buffer (pH 8.0). The samples were then lyophilized for 48 h. The resulting dried powders obtained were labelled as pH tuned enzymes [10].

### Ultrasonic treatment

Ultrasonic treatment was performed using an Elma transsonic digital ultrasonic unit (model T 490 DH) from Elma & Co. KG Hans Schmidbauer Gmbh, Singen, Germany. A fixed frequency of 40 kHz and various power ratings specified in the figure legends were employed. The ultrasonicator bath was equipped with a temperature control.

### The effect of ultrasonic pre-irradiation on esterase activity

pH-tuned lipases (1 mg), each dispersed in organic solvents (100 μl) or dissolved in 20 mM of phosphate buffer (pH 7.0) (100 μl) were put into a microcentrifuge tube (1.5 ml capacity). The lipases were ultrasonicated periodically at various power ratings (110, 66, 44 W) for different time periods (i.e. 1, 2, 3 and 4 h). To avoid substantial rises in temperature, ultrasonication of cells or enzymes was not carried out continuously. Instead an 'on' and 'off' cycle was followed. In this study, we carried out ultrasonication for 5 minutes, followed by a break of 10 minutes between each cycle. This ensured that the temperature of the sample could be kept constant at 40 ± 1°C throughout. After ultrasonic irradiation, the enzyme suspensions (in organic solvents) were centrifuged at 8,000 g for 10 min at 25°C. The supernatants were removed and the lipases dissolved in 1 ml of 0.1 M sodium phosphate buffer (pH 7.0). An esterase assay using *p*-nitrophenyl palmitate as a substrate was performed at 37°C [25]. One unit of enzyme activity corresponds to the production (by 1 mg of lipase) of 1 μmol of *p*-nitrophenol per minute [25]. All the reactions were carried out in duplicate and the difference in the esterase activity value for each pair of duplicates was less than 6%.

### Preparation of enzyme precipitated and rinsed with propanol (EPRP) [26] with ultrasonically pre-irradiated lipase

To dry, chilled acetone (4 ml) at 4°C was added ultrasonic pre-irradiated lipase (10 mg) in 1 ml of 20 mM phosphate buffer (pH 7.0). The precipitate was twice rinsed with 1 ml of ice-cold acetone at 4°C. Prior to use, EPRP was twice washed with 1 ml of *n*-octane. As a control, EPRP of untreated lipase was prepared in a similar manner [26].

### Transesterification of ethyl butyrate with butanol

Ethyl butyrate (60 mM) and *n*-butanol (120 mM) were put into a vial containing 1 ml of octane, before the addition of the lipase EPRP prepared from 10 mg of lipase. The reaction mixture was incubated at 35°C at 200 rpm. Aliquots were withdrawn at different time periods and analysed by GC.

### Ultrasonically pre-irradiated lipase for transesterification of *Jatropha *oil

*Jatropha oil *(0.5 g, 600 μl) and pH tuned lipase (50 mg) were sonicated in a screw-capped vial at 110 W for different time periods (i.e. 1, 2, 3 and 4 h). In this study, we carried out ultrasonication for 5 minutes, followed by a break of 10 minutes between each cycle. This ensured that the temperature of the sample could be kept constant at 40 ± 1°C throughout. Ethanol (137 μl) was added to the ultrasonically pre-irradiated *Jatropha *oil/lipase mixture, and incubated at 40°C with constant shaking at 200 rpm for 24 h. The reaction was monitored by withdrawing aliquots at different time intervals and analysing them using GC. For analysis, the mixture was centrifuged and aliquots were withdrawn from the clear supernatant and diluted (5 times) with hexane. Lauric acid methyl ester was used as an internal standard. All the reactions were carried out in duplicate and the yields obtained from duplicate pairs were found to be within 5% agreement.

### Gas chromatography analysis

The formation of alkyl esters was analysed on a Nucon-5700 gas chromatograph with a flame-ionisation detector. A capillary column (70% phenyl polysilphenylenesiloxane) was employed (length: 30 m; internal diameter: 0.25 mm). Nitrogen was used as the carrier gas at a constant flow rate of 4 Kg cm^-2^. The column oven temperature was programmed from 150 to 250°C (at the rate of 10°C min^-1^) with injector and detector temperatures set at 240 and 250°C, respectively.

### Purification of *Burkholderia cepacia *lipase

*Burkholderia cepacia *lipase was purified by gel filtration as described by Kim *et al *[27]. A sample of crude lipase (2 g) was dissolved in 20 ml of 5 mM phosphate buffer (pH 7.0). To remove insoluble material, the crude enzyme solution was centrifuged at 8000 g for 20 min at 4°C. The supernatant was centrifuged again at 11,000 g for 30 min at 4°C. The clear supernatant was freeze-dried at -20°C and lyophilised for 24 h. 200 mg of the crude enzyme powder was then dissolved in 0.5 ml of 50 mM phosphate buffer and loaded on a Sephadex G-75 column (18.5 cm × 1.75 cm), which had been previously equilibrated with 5 mM of phosphate buffer (pH 7.0). Elution was performed using the same buffer. SDS/PAGE of the sample was performed as described by Hames [28] using a Bio-Rad Mini Orotean II electrophoresis unit and standard molecular-mass markers (Bio-Rad, Richmond, CA, USA). The gel was stained according to the silver staining method [28]. The purified lipase was seen as a single band in SDS-PAGE.

### Circular Dichroism (CD)

Far-UV CD spectra were recorded on a JASCO J-710 spectropolarimeter at 25°C over the 195–250 nm spectral range, with an optical path of 0.2 cm. The lipase concentration was 0.01 mgml^-1 ^of 20 mM phosphate buffer (pH 7.0). Near-UV spectra were recorded over the 250–310 nm spectral range, with an optical path of 0.2 cm and lipase concentration of 1 mgml^-1 ^of 20 mM phosphate buffer (pH 7.0).

### Scanning Electron Microscopy (SEM)

SEM of samples was carried out on a Cambridge Stereoscan (S-360), Altran Corporation, Boston, USA. All the samples were dissolved in 0.1 M phosphate buffer (pH 7.0), freeze-dried and placed on the sample holder before being scanned under vacuum.

## Authors' contributions

SS performed all experiments, drafted sections on materials and methods, and produced figures. MNG participated in the experimental design, discussions, interpretation of results and drafting the manuscript text. Both authors approved the final version.
